# Lipid quantitation and metabolomics data from vitamin E-deficient and -sufficient zebrafish embryos from 0 to 120 hours-post-fertilization

**DOI:** 10.1016/j.dib.2017.02.046

**Published:** 2017-02-21

**Authors:** Melissa McDougall, Jaewoo Choi, Hye–Kyeong Kim, Gerd Bobe, J. Frederik Stevens, Enrique Cadenas, Robert Tanguay, Maret G. Traber

**Affiliations:** a Linus Pauling Institute, Oregon State University, Corvallis, OR 97331, USA; b College of Public Health and Human Sciences, Oregon State University, Corvallis, OR 97331, USA; c The Catholic University of Korea, Seoul, Republic of Korea; d College of Pharmacy, Oregon State University, Corvallis, OR 97331, USA; e Environmental Health Sciences Center, Oregon State University, Corvallis, OR 97331, USA; f University of Southern California, School of Pharmacy, Los Angeles, CA 90089, USA; g Environmental and Molecular Toxicology, Oregon State University, Corvallis, OR 97331, USA; h Sinnhuber Aquatic Research Laboratory, Oregon State University, Corvallis, OR 97331, USA

**Keywords:** Alpha-tocopherol, Docosahexaenoic acid, Choline, Glucose, Ferroptosis

## Abstract

The data herein is in support of our research article by McDougall et al. (2017) [1], in which we used our zebrafish model of embryonic vitamin E (VitE) deficiency to study the consequences of VitE deficiency during development. Adult 5D wild-type zebrafish (*Danio rerio*), fed defined diets without (E–) or with VitE (E+, 500 mg *RRR*-α-tocopheryl acetate/kg diet), were spawned to obtain E– and E+ embryos that we evaluated using metabolomics and specific lipid analyses (each measure at 24, 48, 72, 120 hours-post-fertilization, hpf), neurobehavioral development (locomotor responses at 96 hpf), and rescue strategies. Rescues were attempted using micro-injection into the yolksac using VitE (as a phospholipid emulsion containing d_6_-α-tocopherol at 0 hpf) or *D*-glucose (in saline at 24 hpf).

**Specifications Table**

**Table:** 

Subject area	*Biology*
More specific subject area	*Nutrition; Antioxidants*
Type of data	*Graphs; Figures*
How data was acquired	*LC-MS (liquid chromatography-mass spectrometry) using a Shimadzu Nexera system (Shimadzu; Columbia, MD, USA) coupled to a high-resolution hybrid quadrupole–time–of-flight mass spectrometer (TripleTOF*® *5600; SCIEX; Framingham, MA, USA); Embryos were assessed for viability, developmental progression and spontaneous movements (earliest behavior in zebrafish), using the zebrafish acquisition and analysis program (ZAAP). Locomotor Response Assay using a Viewpoint ZebraBox (software version 3.0, Viewpoint Life Sciences, Lyon, France).*
Data format	*Analyzed*
Experimental factors	*Adult 5D zebrafish fed defined diets without (E–) or with VitE (E+, 500 mg RRR-α-tocopheryl acetate/kg diet) were spawned to obtain E– and E+ embryos that were evaluated up to 120 hours-post-fertilization (hpf)*
Experimental features	*Lipid and metabolomics analyses were performed in developing E– and E+ zebrafish embryos collected daily from 24 to 120 hpf. Mortality and neurobehavioral outcomes were assessed at 96 hpf in two separate rescue experiments using E– and E+ embryo populations micro-injected into the yolk (1) at 0 hpf with VitE or (2) at 24 hpf with D-glucose*
Data source location	*Oregon State University, Corvallis, OR 97330*
Data accessibility	*Data is within this article*

**Value of the data**

• Fatty acid quantification and peroxidation data during zebrafish embryonic development in E- vs. E+ zebrafish embryos may be used by other researchers to investigate antioxidant effects of VitE with respect to specific lipids.
• The metabolomics dataset may be utilized by other researchers to investigate the secondary metabolic effects of VitE deficiency.
• Rescue studies using microinjection into the yolksac may be compared to other methods of compound/nutrient delivery to developing zebrafish.

## 1 Data

**Fig. 1:**
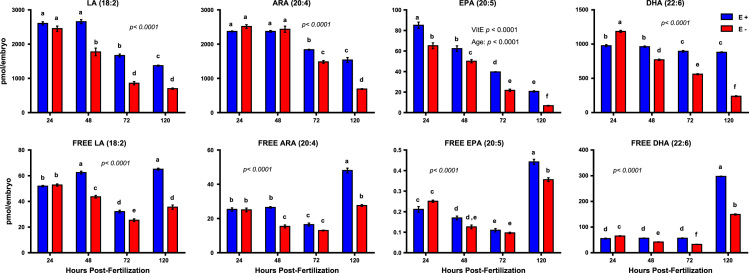
Quantified levels of total and free (unesterified) fatty acids in E– vs. E+ embryos. Area counts normalized using internal standards (*n*=3 samples/group, with *n*=10–15 embryos/sample for total lipids; *n*=4 samples/group with *n*=15-30 embryo/sample for free fatty acids). Shown are saponified (upper row) or extracted only (lower row) samples, means±SEM; *p-*values are for VitE x Age interactions, unless main effects (VitE or Age) are indicated (Tukey’s post-test, *p*<0.05 for bars bearing different letters). Abbreviations: LA (linoleic acid); ARA (arachidonic acid); EPA (eicosapentaenoic acid); DHA (docosahexaenoic acid).

**Fig. 2:**
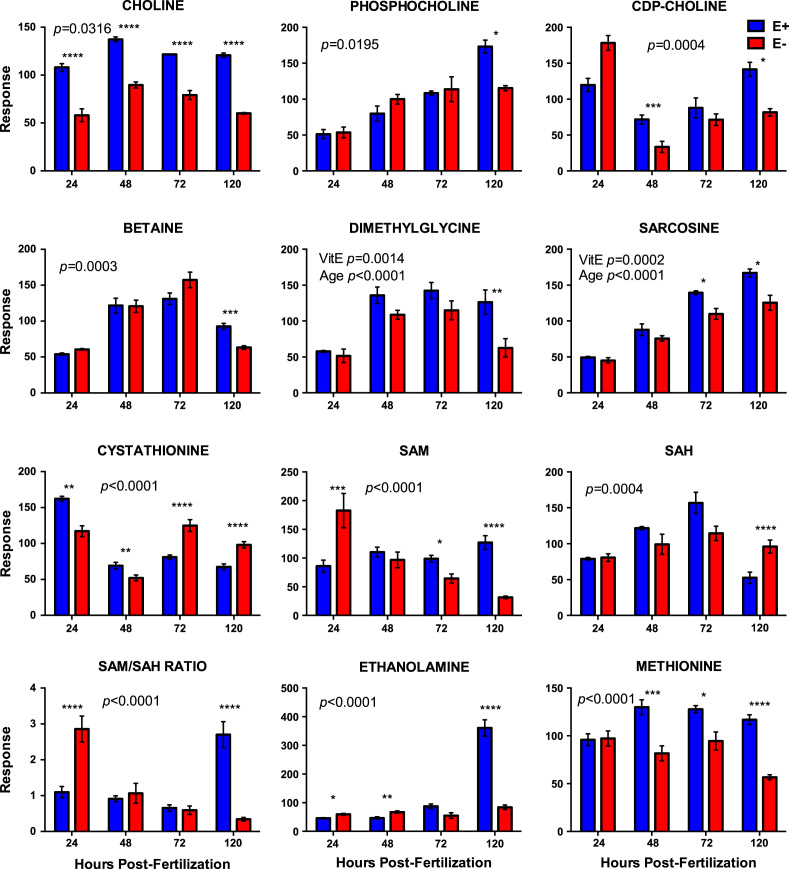
Relative response intensities of choline and methylation pathway intermediates. E– and E+ embryo (*n*=15/sample; 4 samples/group) data were normalized against QC sample intensities (*n*=4) for each individual metabolite. Statistical significance (*p*<0.05) was calculated using 2-way ANOVA with Sidak’s post-test for multiple comparisons of normalized and natural log-transformed intensity values. Shown are means ± SEM; *p-*Values are for VitE x Age interactions, unless indicated otherwise. Paired comparison, p-values are indicated as *<0.05, **<0.005, ***<0.001, **** <0.0001.

**Fig. 3:**
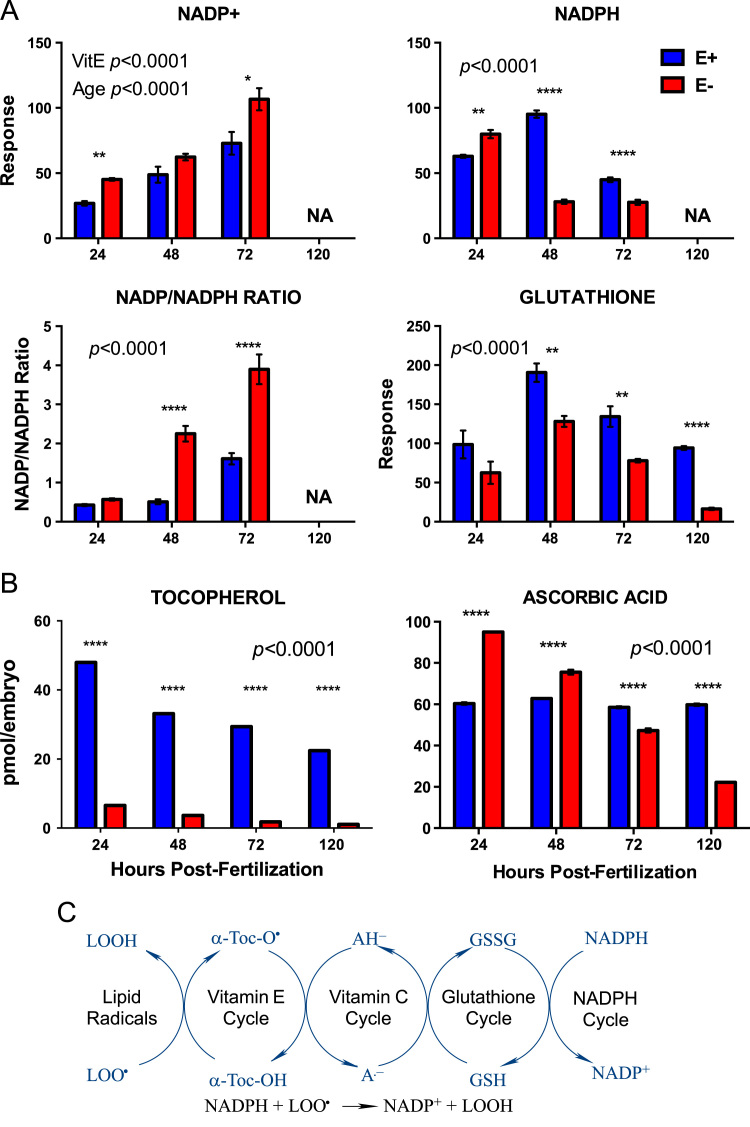
Relative response intensities of antioxidant network components from metabolomics and quantification of α-tocopherol and ascorbic acid. A. E– and E+ embryo (n=15/sample; 4 samples/group) relative response data was normalized against QC sample intensities (*n*=4) for each individual metabolite. B. Quantified levels of α-tocopherol and ascorbic acid, according to established protocols (*31*) and (*33*), respectively. Statistical significance (*p*<0.05) was calculated using 2-way ANOVA with Sidak’s post-test for multiple comparisons of normalized and natural log-transformed intensity values. Shown are means ± SEM; *p-*Values are for VitE x Age interactions, unless indicated otherwise. Paired comparisons *p*-values are indicated as *<0.05, **<0.005, ***<0.001, **** <0.0001. C. Antioxidant network scheme showing interaction of antioxidants with lipid radicals and consumption or NADPH.

**Fig. 4:**
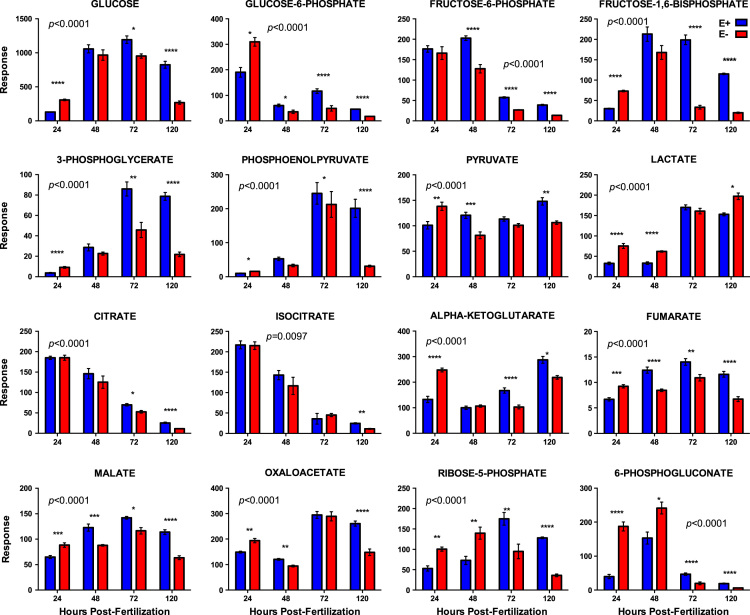
Relative response intensities of glycolytic and tricarboxylic acid cycle intermediates. E– and E+ embryo (*n*=15/sample; 4 samples/group) data were normalized against QC sample intensities (*n*=4) for each individual metabolite. Statistical significance (*p*<0.05) was calculated using 2-way ANOVA with Sidak’s post-test for multiple comparisons of normalized and natural log-transformed intensity values. Shown are means ± SEM; *p-*values are for VitE x Age interactions. Paired comparisons p-values are indicated as *<0.05, **<0.005, ***<0.001, **** <0.0001.

**Fig. 5:**
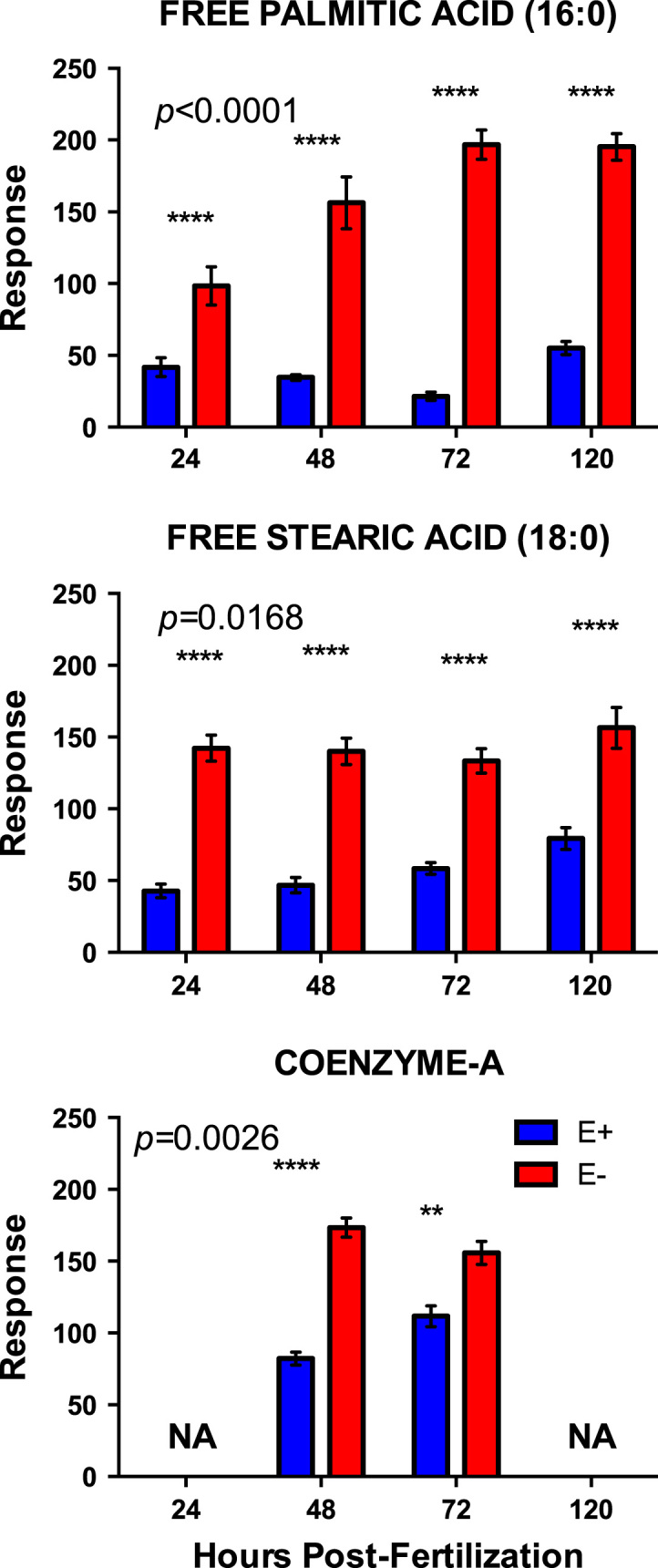
Relative response intensities of free saturated fatty acids and coenzyme A from metabolomic analyses. E– and E+ embryo (*n*=15/sample; 4 samples/group) data were normalized against QC sample intensities (*n*=4) for each individual metabolite. Statistical significance (*p*<0.05) was calculated using 2-way ANOVA with Sidak’s post-test for multiple comparisons of normalized and natural log-transformed intensity values. Shown are means±SEM; *p-*values are for VitE x Age interactions. Paired comparison p-values are indicated as *<0.05, **<0.005, ***<0.001, **** <0.0001.

**Fig. 6:**
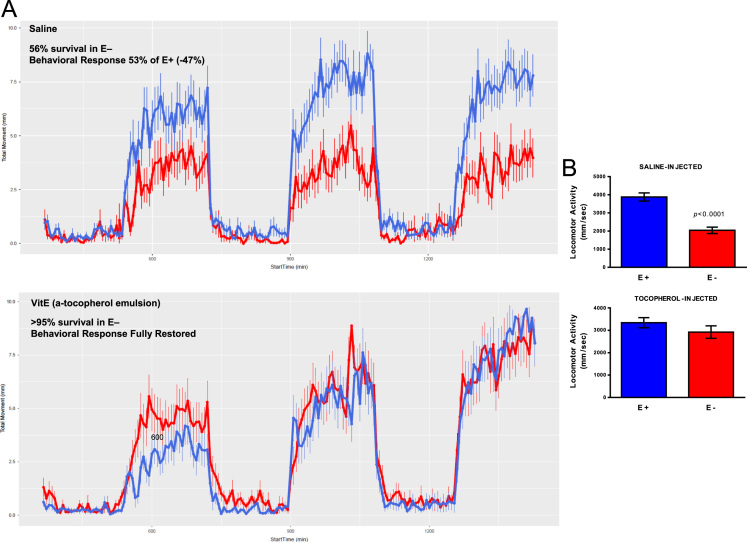
E– compared with E+ embryos have impaired behavior when injected with saline (upper panel), but restored responses when injected with VitE (lower panel). A. Embryos were analyzed in 96-well plates (128 embryos per group). Locomotor activities following a series of light stimuli (a stimulus every 6 for 24 min) were measured as distance moved (mm) over time (seconds). At 96 hpf, E- (red) embryos treated with saline (upper panel) were 47% less responsive to light than were E+ embryos (E– area-under-curve, AUC: 2040±178; E+ AUC: 3877±228; *p*<0.0001). Embryos with morphological defects were not included in data analysis. E- behavior was restored using VitE injection into the yolk at the 1 cell stage (lower panel E– AUC: 2970±280; E+ AUC: 3340±226, not significantly different). B. Bar chart comparisons of respective time-course data. VitE (tocopherol)-injected E– and E+ embryo locomotor activities were not significantly different.

**Fig. 7:**
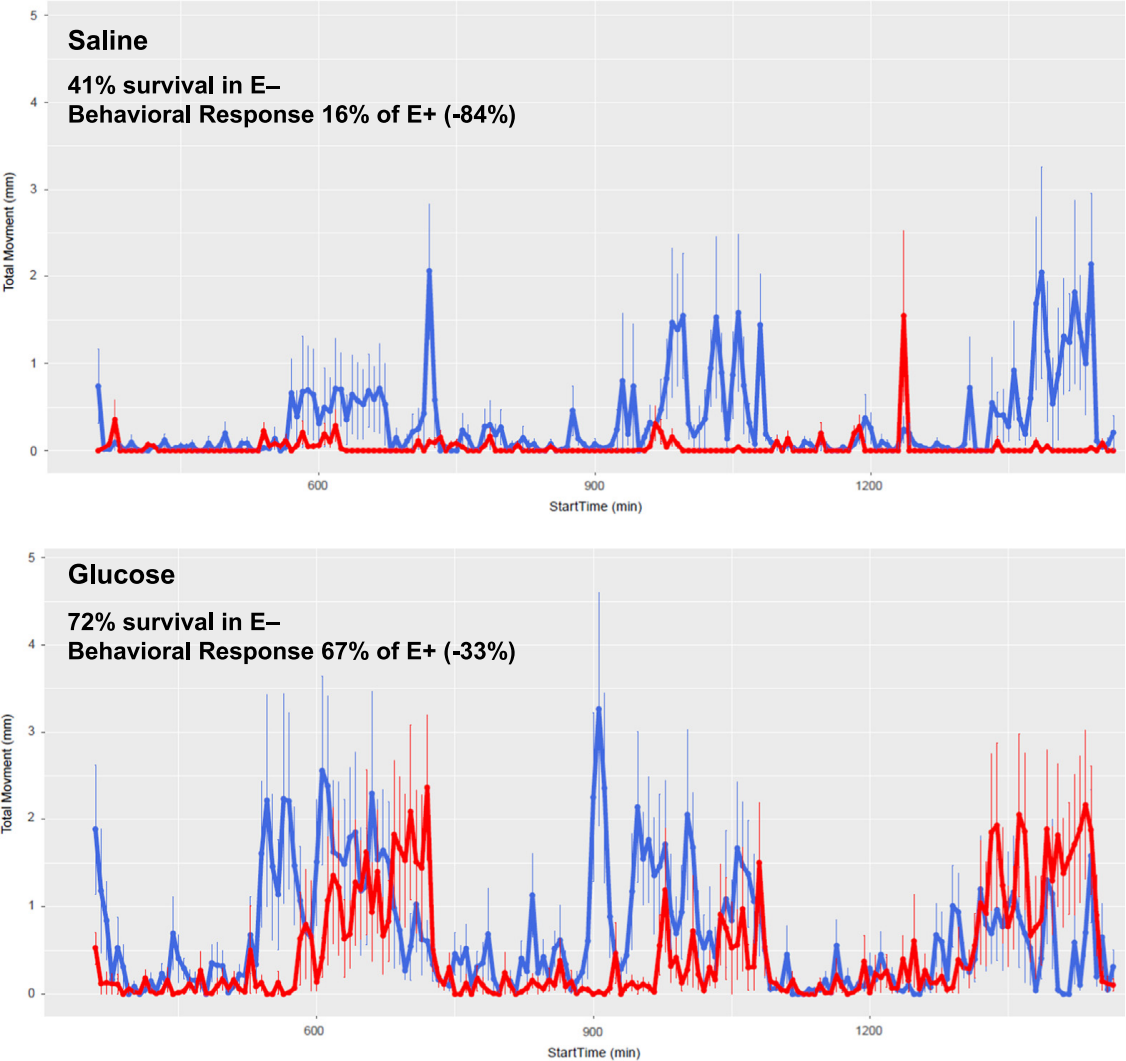
Locomotor response assay activity data showing neurobehavioral impairment. E– and E+ embryos (96 hpf) were analyzed in 96-well plates (128 embryos per group). Locomotor activities following a series of light stimuli (every 6 for 24 min) were measured as distance moved (mm) over time (seconds). E– (red) embryos treated with saline (upper panel) were 84% less responsive to light than were E+ (blue) embryos (E– area-under-curve, AUC: 572±72 E+ AUC: 3580±387; *p*<0.0001). Embryos with morphological defects were not included in data analysis. E– behavior was partially restored by approximately 50% following glucose injection into the yolk at 24 hpf (lower panel; E– AUC: 2502±150; E+ AUC: 3734±359; *p*<0.0001). Statistical significance was determined using a Kolmogorov–Smirnov test (*p*<0.01).

Fig. 1. shows data from quantitative analyses of LA (linoleic acid, 18:2, omega-6); ARA (arachidonic acid, 20:4, omega-6); EPA (eicosapentaenoic acid, 20:5, omega-3); DHA (docosahexaenoic acid, 22:6, omega-3) in fatty acid extracts from samples with and without alcoholic saponification of E– and E+ embryos collected at 24, 48, 72, and 120 hpf. Tables 1and 2 provide detailed targeted metabolomics datasets for E– and E+ embryos collected at 24, 48, 72, and 120 hpf. Relative response intensity metabolomics data for choline and methylation pathway intermediates in E– and E+ embryos are shown in Fig. 2. Relative response intensities of antioxidant network components from metabolomic analyses, as well as quantification of α-tocopherol and ascorbic acid, in E– and E+ embryos (pmol/embryo) are shown in Fig. 3. Relative response intensities of glycolytic and tricarboxylic acid cycle intermediates in E– and E+ embryos are shown in Fig. 4. Relative response intensities of free saturated fatty acids and coenzyme A from metabolomics data in E– and E+ embryo are shown in Fig. 5. Fig. 6 shows locomotor activity data from E– and E+ embryos micro-injected into the yolksac at 0 hpf with either saline or a VitE–emulsion. Fig. 7 shows locomotor activity data from E– and E+ embryos micro-injected into the yolksac at 24 hpf with either saline or *D*-glucose.

## 2 Experimental design, materials and methods

### 2.1 Study design

All experiments (*i.e.* lipid quantifications, targeted metabolomics analyses, and micro-injection rescue studies) were performed in duplicate and have been reported in detail [1].

### 2.2 Zebrafish husbandry and diets

The Institutional Animal Care and Use Committee of Oregon State University approved this protocol (ACUP Number: 4344). Tropical 5D strain zebrafish were housed in the Sinnhuber Aquatic Research Laboratory and complete details of the housing and husbandry have been reported [1].

### 2.3 Analyses

Diet and embryo α-tocopherol [2] and ascorbic acid [3] were determined using high-pressure liquid chromatography with electrochemical detection as reported [1].

Extraction and sample preparation for metabolomic analysis were performed following 24, 48, 72, and 120 hpf, embryos (*n*=15 per replicate, *n*=4 replicates per group), as described [1]. Chromatography was performed with a Shimadzu Nexera system (Shimadzu; Columbia, MD, USA) coupled to a high-resolution hybrid quadrupole–time–of-flight mass spectrometer (TripleTOF® 5600; SCIEX; Framingham, MA, USA). Two different LC analyses using reverse phase and HILIC columns were used, as described [1].

Analysis of total DHA, EPA, ARA, and LA were performed as described [2] with modifications, as described [1]. Chromatographic separations were carried out on 4.6×250 mm J׳sphere ODS-H80 (4 µm, YMC Co, Kyoto, Japan) for negative ion analysis. TOF-MS and TOF-MS/MS were operated with same parameters as for metabolomics, as described [1].

### 2.4 Microinjection rescue studies

Embryos were microinjected as described and criteria used to assess supplementation tolerance of zebrafish embryos using ZAAP at 24, 48, and 120 hpf, as described [1].

### 2.5 Behavioral assessments

Locomotor activity was measured in a total of *n*=128 embryos per group using Viewpoint Zebrabox [4], [5], as described [1].

### 2.6 Data processing and statistical analyses

All data processing and statistical analyses were performed as described in [4], [5], [6], with modification made as reported [1].

## Footnotes

Appendix A Supplementary data associated with this article can be found in the online version at doi:10.1016/j.dib.2017.02.046.
